# Pseudoprogression Following Liver Stereotactic Body Radiotherapy (SBRT) in a Patient With Oligometastatic Leiomyosarcoma: A Case Report

**DOI:** 10.7759/cureus.67835

**Published:** 2024-08-26

**Authors:** Mohamed Aly, Shaheer Shahhat, Timothy K Nguyen

**Affiliations:** 1 Schulich School of Medicine and Dentistry, Western University, London, CAN; 2 Radiation Oncology, London Health Sciences Centre, London, CAN

**Keywords:** radiographic surveillance, gynaecologic oncology, hepatocellular carcinoma, arterial phase hyperenhancement, liver metastasis

## Abstract

Stereotactic body radiotherapy (SBRT) is a non-invasive form of radiation that has been utilized for oligometastatic malignancies. However, pseudoprogression is a common radiological occurrence following this treatment, which manifests as an increase in tumor size before its reduction. We discuss a case of a 58-year-old female patient who initially presented with uterine leiomyosarcoma. Following surgery and postoperative radiation, she was later found to have solitary liver metastasis after three years of surveillance, which was managed by SBRT. However, on short-term follow-up, the lesion was found to have increased in size, prompting discussion regarding whether the growth was a progression of disease or a secondary effect of treatment. After close follow-up, the tumor continued to shrink until it was no longer visible on imaging. This is the first report discussing pseudoprogression following SBRT in a retroperitoneal leiomyosarcoma patient. It serves as a reminder for clinicians to consider the possibility of pseudoprogression before the failure of therapy.

## Introduction

Uterine leiomyosarcoma is a rare, aggressive malignancy most commonly seen in postmenopausal individuals, with an incidence of less than 1% [1,2]. Multimodality treatment is frequently employed for this condition and often includes hysterectomy, systemic therapy, and radiotherapy [3]. The liver is a common site of metastases from leiomyosarcoma, and, in the setting of limited disease, surgical resection of hepatic metastases is an effective treatment approach [4]. There are several options for ablative and localized liver metastasis-directed therapies as well, including doxorubicin-eluting beads (DEB-TACE), yttrium-90 (Y90) radioembolization, and percutaneous microwave ablation [5].

Stereotactic body radiotherapy (SBRT) is an established and effective treatment option for liver metastases [4,6]. However, there is scarce literature on the use of SBRT for liver metastases in leiomyosarcoma. Pseudoprogression is a phenomenon associated with a radiographic increase in initial tumor size after therapy (including systemic and localized therapies), which is followed by stabilization or a reduction in tumor size on subsequent scans [7]. This phenomenon is well characterized in relation to SBRT. We present a case of a 58-year-old female diagnosed with leiomyosarcoma with solitary liver metastasis demonstrating pseudoprogression following SBRT for presumed metastatic uterine leiomyosarcoma. This report is a unique one owing to its description of SBRT for liver metastases of unusual histology and pseudoprogression in the setting of liver SBRT.

## Case presentation

A 58-year-old female presented with a left adnexal mass, which was thought to be benign on initial examination. Carcinoembryonic antigen (CEA) as well as cancer antigen 125 (CA-125) testing was done to rule out ovarian cancer. CEA was within normal limits and CA-125 was only marginally elevated. Due to the location of the mass, preoperative biopsy was not possible. The patient therefore underwent resection of the left pelvic retroperitoneal mass, as well as left and right salpingectomy. Postoperative pathology and CT scan of the chest, abdomen, and pelvis were consistent with a T1b N0 M0 leiomyosarcoma. Surgical margins were negative. Following consultation with Radiation Oncology, she underwent postoperative radiation therapy to a dose of 4500 cGy in 25 fractions to the tumor bed. Adjuvant chemotherapy was not pursued. In the following three years, she underwent surveillance CT imaging of the chest, abdomen, and pelvis every six months, which demonstrated no remarkable changes. 

After three years, one of the surveillance CT scans revealed a 1.6 cm lobulated soft tissue mass within the original operative site, highly concerning for a local tumor recurrence. Tumor board discussions regarding management concurred with repeat surgical resection. In turn, the patient underwent an en-bloc resection of the recurrent disease in the pelvis. The postoperative pathology report described a 2.1 cm mass, consistent with an intermediate-grade leiomyosarcoma. Margins were clear but extended to the peritoneal surface focally.

Follow-up CT demonstrated no additional recurrence in the pelvis but showed an area of hypoattenuation in the hepatic dome. This was further investigated with an MRI of the abdomen, which demonstrated a likely metastatic tumor measuring 2.9 x 1.8 cm in segment 8 of the liver. At the multidisciplinary tumor board discussion, the case was assessed as oligometastatic based on available imaging, and recommendations were made to pursue aggressive treatment. Surgical resection was discussed but there was hesitancy given the rapid recurrence following the previous surgery. Therefore, the recommendation was to offer the patient SBRT to the solitary liver metastasis, which the patient subsequently accepted. 

**Figure 1:**
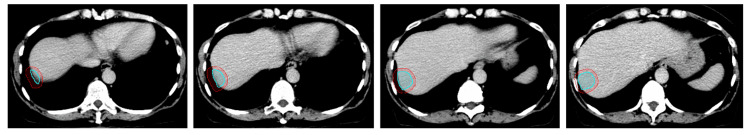
CT images demonstrating radiographic changes in tumor appearance: pre-treatment 4DCT simulation with intravenous and oral contrast Blue: gross tumor volume (GTV); red: planned treatment volume (PTV) CT: computed tomography

The patient was treated with volumetric modulated arc radiotherapy (VMAT) to a dose of 6000 cGy in five fractions every other day using 6 megavolt photons. The patient was simulated using 4D CT with IV contrast and an MR simulation scan with gadolinium fused to guide target delineation. Treatment was delivered with wide-amplitude gating with the patient free-breathing. Gross tumor volume (GTV) consisted of the gross tumor appreciated on all simulation imaging. No clinical target volume (CTV) was generated. The planning treatment volume (PTV) was a 5 mm isotropic expansion on the GTV. The GTV and PTV as they appeared on the patient's simulation scan are shown in Figure 1. The PTV received at least 6000 cGy with the GTV receiving a dose over 6000 cGy (up to a max point dose of 6954 cGy). Image guidance was achieved using daily cone beam CT (CBCT) scans in the treatment unit. Treatment was well tolerated and no acute toxicities were reported by the patient.

**Figure 2:**
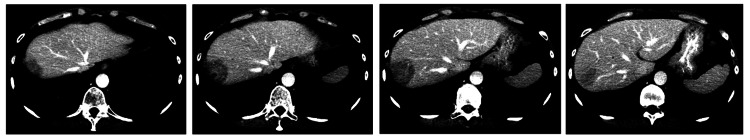
CT images demonstrating radiographic changes in tumor appearance: CT with intravenous and oral contrast 4.5 months post-treatment CT: computed tomography

In the first CT follow-up (six weeks post-treatment), there was no change in size, but the lesion was more hypoattenuating in the portal venous phase (suggesting early treatment response). On a subsequent three-month follow-up CT scan (i.e. 4.5 months post-treatment), the lesion had increased in size from its original 2.9 x 1.8 cm to 4.3 x 4.0 cm. The enhancement pattern was similar to the previous six-week scan. The increased lesion size as it appeared on the patient's CT scan is shown in Figure 2.

This increase in size and persistent enhancement raised suspicion regarding early disease progression and was reported as such on the imaging report. However, when the imaging changes were compared against the previous treatment volume, it was apparent that the enlarged tumor was still encompassed by the previous high dose volumes (90%). These findings raised the possibility of pseudoprogression rather than true progression. After discussions with the multidisciplinary team and the patient, the decision was made to request a short-interval CT scan in six weeks for a reassessment.

**Figure 3:**
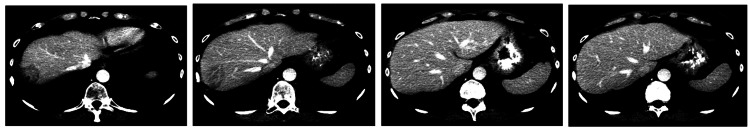
CT images demonstrating radiographic changes in tumor appearance: CT with intravenous and oral contrast six months post-treatment CT: computed tomography

The follow-up CT scan six weeks later demonstrated a decrease in tumor size, from 4.3 x 4.0 cm to 3.8 x 3.6 cm, and an unchanged pattern of enhancement. This was reassuring and suggestive of radiation-related effects rather than true progression. In keeping with this assessment, subsequent CT scans at three-month intervals revealed a continual reduction in lesion size until it was no longer radiographically visible 11 months post-treatment. The shrunken lesion as it appeared on the scan is shown in Figure 3.

The patient recently completed her 36-month follow-up and continues to do well without any radiographic or clinical signs of recurrence.

## Discussion

In this report, the increase in the size of the treated liver lesion in combination with persistent enhancement on imaging raised suspicion for early tumor progression and was identified as such on the radiology report. Close imaging surveillance every three months was reassuring, demonstrating a pattern consistent with pseudoprogression. This report highlights the importance of the treating radiation oncologist remaining involved in the case after treatment to lend their expertise and also review the treatment plan to compare treated volumes and changes on follow-up scans. It is also important for all members of the care team to be aware of this potential phenomenon. Electing for a subsequent short-interval scan may help establish a diagnosis and avoid unnecessary, premature interventions, or changes in management. In this case, ongoing close surveillance revealed a reduction in size and enhancement on subsequent imaging, confirming a diagnosis of pseudoprogression. 

Pseudoprogression is an increase in the size of a tumor lesion and/or changes in imaging characteristics that mimic tumor progression but are related to evolving treatment effects. It is a well-understood phenomenon concerning malignant tumors treated with SBRT across many histologies and anatomical sites. Zhang et al. demonstrated that six out of 121 glioma patients treated with radiotherapy showed pseudoprogression [8]. Another case report demonstrated pseudoprogression of lymph node metastasis following SBRT in a patient with esthesioneuroblastoma [9]. Studies in patients with brain metastases have reported that pseudoprogression is common, with the incidence ranging from 13 to 32% [10]. Similarly, rates of pseudoprogression after spine SBRT were about 18% [11].

The use of liver SBRT is a well-established treatment for hepatocellular carcinoma (HCC) and liver metastases [6,12]. Following SBRT for HCC, a combination of factors has been validated as a tool to assess tumor response to the procedure, including change in target lesion size, change in internal enhancement characteristics, washout appearance, and an estimation of necrosis [12,13]. Post-treatment imaging algorithms typically use enhancement and lesion size progression as key indicators for viable disease. Concerning size, irradiated liver lesions have been noted to either remain the same size or decrease in size on post-treatment follow-up imaging [14-16]. An increase in the size of a previously treated liver lesion has been cited as highly suspicious for local recurrence [17]. The majority of treated HCCs will exhibit arterial phase hyperenhancement for three months or more and thus residual enhancement is not a reliable predictor alone [17]. Additionally, a reduction of liver tumor enhancement has been found to precede a reduction in tumor size [13].

Pseudoprogression changes in imaging can happen acutely (<3 months follow-up), subacutely (three to six months), or chronically (>6 months); and in each setting, there are unique imaging characteristics [13]. There is evidence that imaging changes lag behind objective tumor response. Sanuki et al. demonstrated that 76% of SBRT-treated HCC patients showed enhancement in imaging three months after therapy and even 29% at 12 months [18]. The authors demonstrated that in some tumors, even after complete tumor response, there remained evidence of tumor vascularity up to 12 months after SBRT [18]. Kimura et al. demonstrated that 25% of their patient cohort had residual arterial hyperenhancement at three months, but declined to 2% at six months post-radiation [19]. The study noted that, when the residual hyperenhancement for more than three months was perceived as local progression, the treatment results differed significantly [19]. These studies demonstrate that pseudoprogression is a common phenomenon after liver SBRT.

Due to the prevalence of pseudoprogression, new conclusions have been reached regarding management. Arterial phase hyperenhancement within the HCC region and surrounding off-target hepatic tissue is common if imaging is performed less than three months after radiation therapy, which may interfere with clinician interpretation of tumor response [17]. It appears that the optimal time for an initial assessment after treatment is at least 6-12 months, and arterial phase hyperenhancement may persist even with a complete pathologic response [20]. Changes in our patient’s lesion are described in follow-up after more than three months. This supports that, while the window for pseudoprogression is wide, attentiveness to this phenomenon during the 6-12 months may be particularly important. 

To our knowledge, this is the first report of pseudoprogression of solitary liver metastasis of leiomyosarcoma following SBRT. There are certain limitations worth considering in this report. Inherently, case reports may be different from other clinical case-related studies. Additionally, leiomyosarcoma is an uncommon diagnosis, which also limits the generalizability of these findings. Further research examining the imaging characteristics of liver metastases of differing histologies would contribute immensely to the literature on the subject.

## Conclusions

This report highlights the favorable outcomes in a unique case involving liver SBRT for oligometastatic leiomyosarcoma and emphasizes the importance of considering pseudoprogression during imaging surveillance. This report is significant as it is the first of its kind demonstrating pseudoprogression following SBRT in this patient population and highlights the need for a greater understanding of pseudoprogression among clinicians. Further study of the nature and imaging characteristics of pseudoprogression following liver SBRT for metastases of different histologies would lead to a better understanding of this phenomenon and its implications on patient care.
